# Evaluation of Cotton (*Gossypium hirsutum* L.) Leaf Abscission Sensitivity Triggered by Thidiazuron through Membership Function Value

**DOI:** 10.3390/plants10010049

**Published:** 2020-12-28

**Authors:** Dingsha Jin, Yanchao Xu, Huiping Gui, Hengheng Zhang, Qiang Dong, Ripon Kumar Sikder, Xiangru Wang, Guozheng Yang, Meizhen Song

**Affiliations:** 1 State Key Laboratory of Cotton Biology, Institute of Cotton Research, Chinese Academy of Agricultural Sciences, Anyang 455000, China; jindingsha@caas.cn (D.J.); xuyc@cricaas.com.cn (Y.X.); guihuiping@caas.cn (H.G.); zhanghengheng@caas.cn (H.Z.); dongqiang@caas.cn (Q.D.); 2017Y90100144@caas.cn (R.K.S.); 2 MOA Key Laboratory of Crop Eco-Physiology and Farming System in the Middle Reaches of Yangtze River, College of Plant Science and Technology, Huazhong Agricultural University, Wuhan 430000, China; 3 School of Agricultural Sciences, Zhengzhou University, Zhengzhou 450001, China

**Keywords:** cotton, leaf abscission, thidiazuron, MFV, principal component weighting

## Abstract

Chemical defoliation is an essential agricultural practice in cotton production for mechanic harvesting. Thidiazuron (TDZ) is the active ingredient of the chemical defoliant used on cotton. So far, few studies havefocused on the method of identifying the sensitivity of cotton cultivars to TDZ. Therefore, a greenhouse soil culture experiment was performed by using two widely cultivatedupland cotton cultivars CRI 49 and CRI 12 treated with seven different concentrations (0, 100, 200, 300, 400, 500, and 1000 mg L^−1^) of TDZ at the seedling stage to establish a screening system. Principal component analysis and the membership function value (MFV) method was used to analyze the physiological and phenotypic characters, including abscission rate, amino acids content, net photosynthetic rate (Pn), etc. Finally, we developed a mathematical evaluation model, selected 100 mg L^−1^ TDZ as the optimal concentration and identified reliable characters net photosynthetic rate (Pn), stomatal conductance (Gs), and transpiration rate (Tr) to evaluate cotton leaf abscission sensitivity. These results also confirmed that CRI 12 was more sensitive to TDZ than CRI 49. This is the first time using a mathematical evaluation method to evaluate the cotton leaf abscission sensitivity triggered by TDZ at the seedling stage and the results were also confirmed in the field experiment. Furthermore, it will be valuable that MFV method is applied to stress sensitivity evaluation in other crop species under stress environment.

## 1. Introduction

Abscission of plant parts, such as leaves, flowers, or fruit, is a fundamental biological process of plants sin owing to biotic or/and abiotic stress [1,2,3], natural aging, or periodic developmental events [4,5]. Premature abscission of flowers and fruits reduces crop yield in the field [2]. In general, a lower abscission rate is beneficial for the economic benefit of crops due to improving yields and quality of crop [3]. However, leaf shedding is conducive to mechanized harvesting in cotton [6,7] and sugarcane [8,9]. Therefore, chemical defoliation is an essential agricultural practice in cotton production. Uniform spraying of chemical agent, which gives rise to defoliation at the proper time before harvesting, can improve the defoliation efficiency and decrease the trash content of cotton [6,10,11]. The active ingredient of the frequently-used chemical defoliator is thidiazuron (TDZ) which is a kind of cytokinin analogues [12,13,14]. A previous study found that leaf shedding induced by TDZ is due to enhancing the synthesis of endogenous ethylene [6,13,15], which could destroy the polar transport of auxin [14] and increase the activity of endogenous cell wall hydrolase in the abscission zone (AZ) in cotton [16]. Our previous study showed that Reactive oxygen species (ROS) and photosynthesis synergically regulate leaf abscission of TDZ treatment in cotton [17]. Cotton defoliation efficiency with TDZ treatment depends on leaf abscission sensitivity, which varies between cultivars as well as TDZ dosage. In response to TDZ, the sensitive cultivar produced early abscission zone and has higher defoliation rate than the insensitive cultivar [6].

So far, there is a lack of quantitative evaluation method for abscission sensitivity in cotton against TDZ treatment.The fuzzy comprehensive evaluation method has been applied to plant stress researches [18,19], and the method transforms qualitative evaluation into quantitative evaluation according to the membership theory of fuzzy mathematics. Membership function value (MFV) analysis was used to evaluate salt tolerance germplasm, selecting the optimal salt concentration and the reliable salt tolerance indexes in *Brassica napus* [20], *Gossypium hirsutum* L [21], and *Sorghum bicolor* (L.) [22]. MFV analysis was also applied to assess drought tolerance in *Triticum aestivum* L. [23] and Millet Germplasm (*Panicum miliaceum* and *Setaria italica*) [19]. In the current study, MFV analysis was applied to understand the sensitivity of cotton cultivars to defoliation against TDZat the seedling stage. Though, the accuracy of the evaluation results mainly depends on the method of weighting. Thus, principal component weighting (PCW) method is firstly used to conduct comprehensive evaluation analysis of cotton leaf abscission sensitivity.

The comprehensive evaluation analysis of cotton leaf abscission sensitivity is dependent on environmental factors and cotton growth stages. To avoid the influence of aging and other environmental factors, TDZ treatment was applied to cotton plants at the seedling stage in the greenhouse. Subsequently, a field experiment was carried out to verify the reliability of the results obtained from the seedling stage experiment. We investigated leaf phenotype characteristics, amino acids, soluble sugar, photosynthetic pigment, and photosynthetic parameters under different concentrations of TDZ in the greenhouse and leaf abscission rate in the field. Our previous study proved that these indicators are mirrored to leaf abscission to some extent. The overall objectives of the current study were to (1) screen the optimum TDZ concentration at the seedling stage; (2) identify evaluation indexes of leaf abscission sensitivity; and (3) evaluate the leaf abscission sensitivity of CRI 49 and CRI 12 under both greenhouse and field conditionn. Our research provided a reference method to evaluate abscission sensitivity and an available system to study the mechanism of leaf abscission, as well as othertissue abscission.

## 2. Results

### 2.1. Comprehensive Evaluation of Principal Component Weighting

**Figure 1:**
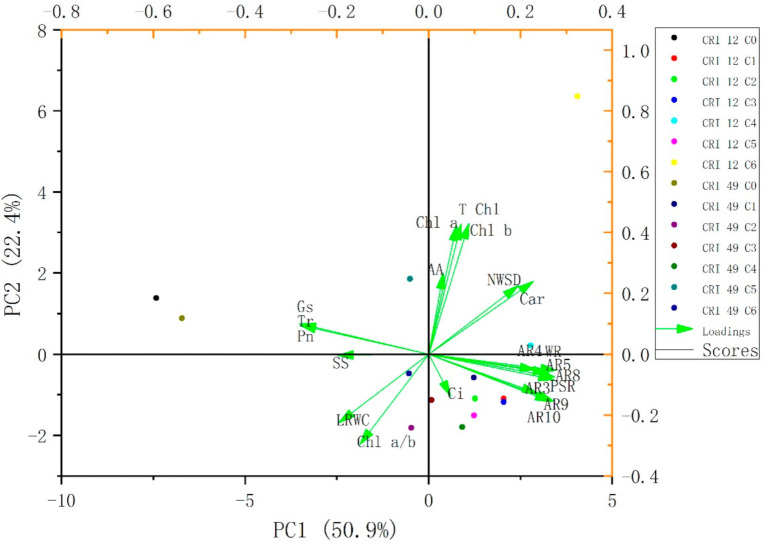
Cluster analysis of different concentration thidiazuron (TDZ) treatment in CRI 49 and CRI 12.

To understand the contribution degree of leaf abscission sensitivity evaluation indexes, principal component analysis (PCA) was used to weight the selected indicators for the first time. PCA clustering analysis showed that the TDZ treatments were significantly distinguished from the control treatment (Figure 1). Tr, net photosynthetic rate (Pn), Gs, soluble sugar (SS), leaf relative water content (LRWC), and the ratio of chlorophyll a and chlorophyll b (Chl a/b) was negatively correlated with leaf abscission rate (AR), while other indicators were positively correlated with AR (Figure 1).

Five principal components (PC 1, PC 2, PC 3, PC 4, and PC 5) could be utilized to explain the difference between genotypes and treatments in 21 indexes, and the cumulative contribution rate reached 91% (Table 1). The PC 1 represented 50.9% of the variability primarily for leaf abscission (AR;3-10 DAT), carotenoid (Car), leaf relative water content (LRWC), natural water-saturated deficit (NWSD), SS, wilting incidence (WI), purple spot incidence (PSI), Tr, Pn, and Gs were the main indexes (Table 1). The contribution rate of the PC 2 was 22.4%, and the main indexes were chlorophyll a (Chl a), chlorophyll b (Chl b), and total chlorophyll (T Chl) (Table 1). The contribution rate of PC 3, PC 4, and PC 5 were nearly 18% (Table 1). In addition to amino acid (AA) and intercellular CO_2_ concentration (Ci), other indicators have a higher weight in 21 indexes, which are an important indicator of cotton leaf abscission after TDZ treatment.

### 2.2. Genotypic Variation and Optimum TDZ Concentration under Leaf Abscission Induced by TDZ Treatment

In this study, two cotton genotypes (CRI 49 and CRI 12) were studied under seven different TDZ concentrations. Based on the selected indexes, a comprehensive evaluation of the genotypes sensitivity and leaf abscission under different concentrations was conducted by MFV method. The results showed the values of fuzzy membership functions and final membership functions (MV) of the two cultivars under different concentrations (Table 2). According to MV, the genotypic difference between CRI 12 and CRI 49 for leaf abscission sensitivity was evaluated, where CRI 12 had higher MV than CRI 49 (Table 2). Therefore, CRI 12 was more susceptible to TDZ induced leaf abscission.

According to the MV, we can evaluate the most confidential treated concentration. The MV for CRI 49 was 0.66, 0.52, 0.62, 0.77, 0.29, and 0.46 and that for CRI 12 was 0.78, 0.65, 0.81, 0.77, 0.69, and 0.53 under 100, 200, 300, 400, 500, and 1000 mg L^−1^ TDZ, respectively (Table 2). The MV was higher under 100 mg L^−1^ and 400 mg L^−1^ TDZ in both genotypes and under 300 mg L^−1^ TDZ only for CRI 12 (Table 2). The MV of 500 mg L^−1^ and 1000 mg L^−1^ TDZ treatment was lower than other treatments, indicating that the high concentration of TDZ treatment was not conducive to leaf abscission (Table 2). Therefore, based on the cost, the 100 mg L^−1^ TDZ can be used as the optimal defoliant concentration.

### 2.3. Evaluation of Leaf Abscission Sensitivity and Screening of Reliable Indexes

The correlation between 13 physiological indexes and leaf abscission sensitivity was established by Pearson’s correlation analysis between 13 physiological indicators and the MV. The results showed that SS, Tr, Pn, and Gs were significantly negatively correlated with MV (Table 3). The indexes of Car, LRWC, and NWSD were highly correlated with MV (Table 3). The indexes of chlorophyll content, AA and Ci had a low correlation with MV (Table 3). Thus,MV can be invoked as the quantization value of leaf abscission sensitivityand SS, Tr, Pn, and Gs were reliable physiological indexes to evaluate leaf abscission sensitivity treated by TDZ.

**Figure 2:**
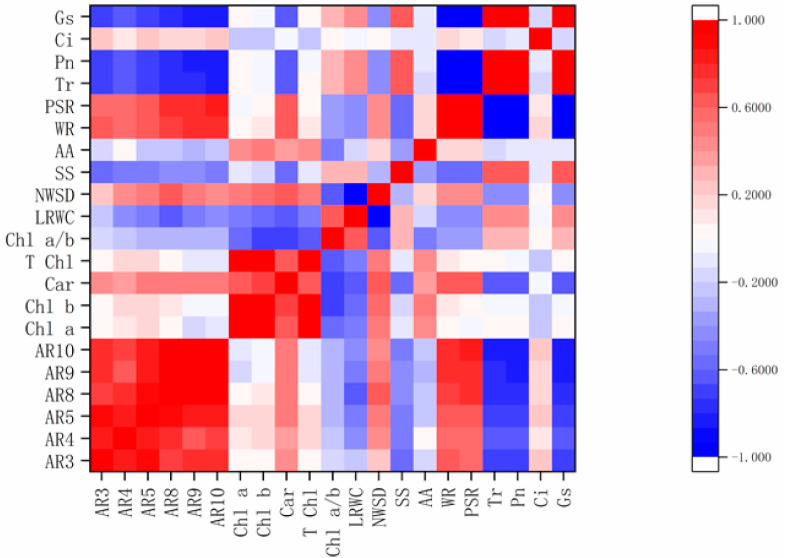
Correlation analysis among different indexes under TDZ treatment. Red and blue colors represent positive and negative correlation, respectively. The darker the color, the higher the correlation. AR3, AR4, AR5, AR8, AR9, AR10 represent leaf abscission rate after treatment at 3, 4, 5, 8, 9, and 10 days, respectively; Chl a, Chlb.,T Chl, Car, Chl a/b, LRWC, NWSD, SS, AA, WI, PSI, Tr, Pn, Ci, Gs represent chlorophyll a, chlorophyll b, total chlorophyll, carotenoid, the ratio of chlorophyll a and chlorophyll b, leaf relative water content, natural water-saturated deficit, soluble sugar, amino acid, wilting incidence, purple spot incidence, transpiration rate, net photosynthetic rate, intercellular CO_2_ concentration, and stomatal conductance, respectively.

We also analyzed the correlation of all morpho-physiological indicators under TDZ treatment by Pearson’s correlation analysis. There was a significant positive correlation among the abscission rates (AR3, AR4, AR5, AR8, AR9, and AR10), however, no significant correlation was found between Chl a, Chl b, T Chl, Chl a/b, LRWC, NWSD, AA, and leaf ARs (Figure 2). Similarly, Car, WI, and PSI were positively correlated with leaf ARs, while SS, Pn, Tr, and Gs have negatively correlated with leaf ARs (Figure 2), suggesting that these indexes can be predicted as the phenotype and physiological indicators, especially Pn, Tr, and Gs.

### 2.4. Effects of Different TDZ Concentrations on Leaf Abscission and Photosynthetic Characteristics

**Figure 3:**
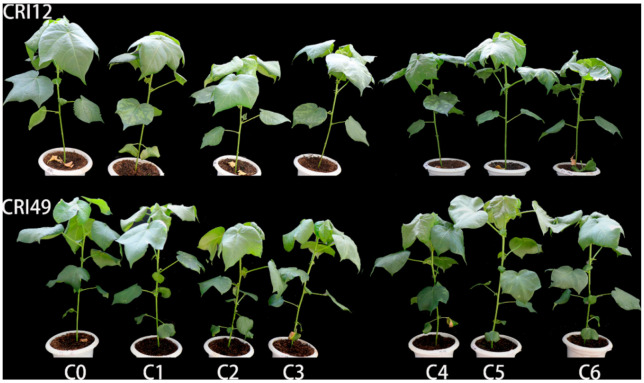
Phenotype of leaf abscission induced by TDZ (0, 100, 200, 300, 400, 500, 1000 mg L^−1^) in CRI 49 and CRI 12 at 2 days after treatment at the seedling stage. Scale bars are 12 cm.

**Figure 4:**
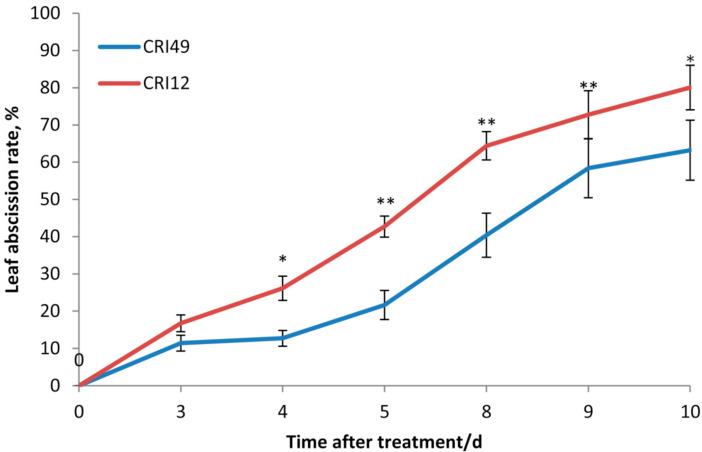
Effects of TDZ on leaf abscission rate between CRI 49 and CRI 12 at the seedling stage. * and ** denote significance at the 0.05 and 0.01 levels, respectively.

The abscission zone of leaves began to start after TDZ application and a concentration of 1000 mg L^−1^ TDZ caused leaf abscission in CRI 12 at 2 DAT (days after treatment) (Figure 3). However, the abscission zone in CRI 49 did not form at the same period of time (Figure 3). The phenotype of purple spots and wilting in the leaves were observed after TDZ treatment (Figure 3). Both CRI 49 and CRI 12 exhibited rapid leaf abscission in response to different concentration of TDZ treatment (Figure 4, Figure S1). The abscission rate of the treatments was higher under 100 mg L^−1^ and 400 mg L^−1^ TDZ in both cultivars (Figure 4). At the same time, it had better defoliation effects under 300 mg L^−1^ TDZ for CRI 12 (Figure 4). Additionally, the higher leaf abscission rate of CRI 12 compared with CRI 49, further indicating that CRI 12 was more susceptible to TDZ than CRI 49 (Figure 4). Therefore, the concentration of 100 mg L^−1^ was the best defoliant concentration. The similar results obtained by the MFV method mean that the evaluation method of MFV is effective and reliable.

In order to ascertain the reliability of screening at the seedling stage, field experiments were also carried out. Leaf abscission rate showed higher after defoliant treatment in both cultivars and regions in the field (Table 4). There was no significant difference between normal and TDZ treatment (Table 4). However, CRI 12 has a higher relative leaf abscission rate than CRI 49 (Table 4). The higher the relative leaf abscission rate, the stronger the leaf abscission sensitivity is. So, the field experiment results further proved that CRI 12 was more sensitive to TDZ than CRI 49, further showing the experimental results at the seedling stage are reliable.

**Figure 5:**
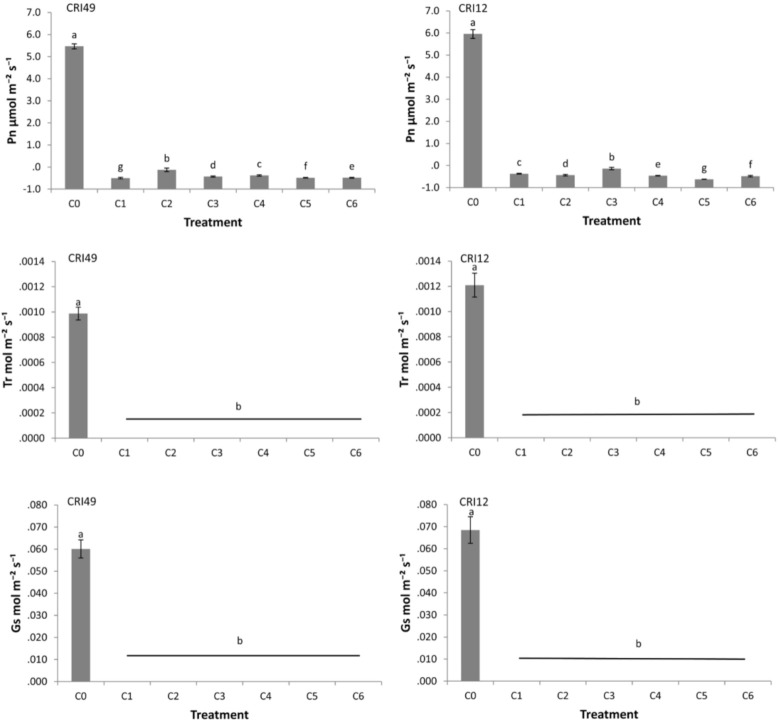
Effects of different concentration of TDZ (C0, C1, C2, C3, C4, C5 and C6: 0, 100, 200, 300, 400, 500, 1000 mg L^−1^) on leaf photosynthetic characteristics at 1 day after treatment at the seedling stage. Pn, Tr, and Gs represent net photosynthetic rate, transpiration rate, and stomatal conductance, respectively. The different letters represent significantly different at *p* = 0.05.

Through the measurement of photosynthetic characteristics 1 day after TDZ treatment, it was found that Pn, Tr, and Gs of leaves all decreased to close to zero values under the treatment of different concentrations of TDZ (Figure 5), indicating that the response of photosynthesis to TDZ was severe. Five hundred and 1000 mg L^−1^ TDZ treatments have the lowest net photosynthetic rate for both cultivars.

## 3. Discussion

The abscission of leaves, flowers and fruit is a natural seasonal phenomenon in plants in the world. Cotton plants show leaf abscission phenomenon due to physiological senescence in the late growth stage and defoliant, in which its major ingredient is TDZ, is applied to cotton production to accelerate leaf shedding process to facilitate mechanical harvesting [7,10]. In the greenhouse, leaf fall off is induced by TDZ at the seedling stage (Figure 4). Plant organ abscission depends on endogenous ethylene increase in the abscission zone [24,25] and cotton leaf abscission triggered by TDZ also is involved with ethylene [6,7]. Ethylene regulating cotton defoliation is confirmed at the molecular level [6]. However, leaf abscission sensitivity varies among different cultivars in cotton [6]. In this study, we studied two cultivars (CRI 49 and CRI 12) and CRI 12 showed higher leaf abscission sensitivity than CRI 49, both at the seedling stage (Table 2, Figure 4) and in the field (Table 4). According to the MV, leaf abscission sensitivity of CRI 12 was higher than that of CRI 49 under different concentrations of TDZ treatment (Table 2). We also find CRI 12 has a higher abscission rate compared to CRI 49 (Figure 4). Phenotypic data and MFV analysis showed that CRI 12 is a defoliation sensitive cultivar compared to CRI 49 at the seedling stage in the greenhouse, and it is further proved in the late growth stage of cotton in the field. Depending on the seedling experiment and field verification, MFV analysis is dependable to access leaf abscission sensitivity.

In our study, the leaf abscission sensitivity evaluation of cotton genotypes and treatments of different concentrations was evaluated by MFV. MFV analysis was used to evaluate salt and drought tolerance in distinct crops [21,22,23]. The accuracy of the evaluation results largely depends on the weighting method and the PCA method is used for determining the contribution rate of the principal components of this study. It is the first time that anyone has developed a mathematical evaluation model using the MFV method to predict the cotton leaf abscission sensitivity induced by TDZ during the seedling stage. The final membership function value (MV) can be utilized to evaluate the most sensitive concentration treatment and the bigger the MV, the higher the leaf abscission sensitivity. The concentrations of 100 and 400 mg L^−1^ have a higher value of MV (Table 2) and cause a higher leaf abscission rate in both cultivars (Figure 4). So, the concentration of 100 mg L^−1^ can be used as the defoliant optimal concentration using a concentration gradient experiment and with the lowest concentration. It was found that TDZ treatment with low concentration had a better effect on leaf shedding, while TDZ treatment with high concentration was not conducive to leaf shedding of cotton (Table 2, Figure 4).

Previous studies have found that TDZ induced leaf abscission by increasing the activity of hydrolytic enzymes of AZ [7], but phenotypic symptoms and physiological change of cotton leaf discs have not been described. In this study, different concentrations of TDZ treatment showed the symptoms of purple spots, wilting (Figure 3), and a huge reduction in Pn, Tr, and Gs to almost zero after one day of TDZ treatment (Figure 5). The phenomenon, in which the leaf wilted, purple spots appeared, and photosynthetic parameters decreased, predate leaf shedding. Wilting and purple spot incidence were positively correlated with leaf abscission rate (Figure 2), indicating wilting and purple spot incidence is a phenotypic prediction before defoliation induced by TDZ. Previous studies have demonstrated that cold stress can halt photosynthesis and induce leaf abscission [26]. Significant reduction of the cotton leaves photosynthesis indicates that TDZ may halt photosynthesis and induce cotton leaf abscission. So, photosynthetic parameters may be important and reliable indexes to evaluate the cotton leaf abscission sensitivity. The correlation analysis among the indicators showed that the Pn, Tr, and Gs of the leaves were negatively correlated with the ARs, carotenoid, wilting and purple spot incidence; however, Pn, Tr, and Gs have shown positive correlation with soluble sugar (Figure 2). Based on the correlation between the physiological indexes and final membership function value (MV), SS and photosynthetic parameters (Pn, Tr, and Gs), were significantly negatively correlated with MV (Table 3), indicating that photosynthetic parameters are reliable indexes to evaluate the cotton leaf shedding sensitivity. Previous studies have concluded that photosynthesis inhibition, associated with nutritional stress, increases flower and fruit abscission [27,28,29]. We speculate that the difference in net photosynthetic rate may result in leaf abscission depending on cultivars sensitivity. However, further studies are necessary to reveal the mechanism of photosynthesis regulating leaf abscission. It is interesting to consider how physiological metabolism and molecular mechanisms respond to TDZ.

## 4. Materials and Methods

### 4.1. Materials

The experiment was carried out in the greenhouse of the cotton research institute of the Chinese academy of agricultural sciences (Anyang, Henan Province, China). The concentration screening and physiological response experiment of leaf abscission induced by TDZ (Shanghai yuanye Bio-Technology Co., Ltd., Shanghai, China) were performed on CRI 12 and CRI 49, which are excellent cotton (*Gossypium hirsutum* L.) cultivars and are widely planted in Xinjiang cotton-growing areas in China. CRI 12 and CRI 49 were also cultivated under normal agricultural conditions in the field in Kuitun (Xinjiang, China) and Anyang (Henan, China) to investigate leaf abscission rate for field verification. Thidiazuron was sprayed using an automatic backpack sprayer when 20 to 40% of cotton bolls were opened (on 16 September 2019 and 5 September 2019).

### 4.2. Experimental Design

A pot experiment was carried out for two biological replicates by using plastic basins (diameter: 12 cm, height: 10 cm) filled with nutritive soil: vermiculite (1:1), 1 plant/basin. The conditions of the greenhouse were set as 16 h light/8 h dark cycles with a temperature of 28 ± 2 °C, 150–250 μmol m^−2^ s^−1^ light intensity, and 50–70% humidity. At the eight leaf stage, healthy and identical seedlings were subjected to treatment through the foliar spray. Seven TDZ concentrations were set in the experiment: 0, 100, 200, 300, 400, 500, 1000 mg L^−1^, and abbreviated as C0, C1, C2, C3, C4, C5, and C6. The experiments were conducted by randomized complete block, three replicates of each treatment and six plants per replicates.

### 4.3. Wilting Incidence, Purple Spot Incidence, and Abscission Rate in the Leaf

Leaves wilting (Lw), leaves with purple spots (Lp), and the total (Lt) were counted and leaf wilting incidence (WI) and purple spot incidence (PSI) were calculated 3 days after treatment (DAT). The remaining leaf number (N) was recorded and the abscission rate (AR3, AR4, AR5, AR8, AR9, and AR10) was calculated at 3, 4, 5, 8, 9, 10 DAT. The plants had been artificially topped, and no new leaves grew. The calculation formula is as follows:

(1) WI=Lw/Lt,

(2) PSI=Lp/Lt,

(3) AR=(Lt−N)/Lt.

### 4.4. Leaf Relative Water Content and Natural Water-Saturated Deficit

Leaf relative water content (LRWC) [30] and natural water-saturated deficit (NWSD) were determined at 3 DAT as follows: Leaf fresh weight (FW) were weighted, then soaked in distilled water until saturated weight (SW), and, finally, dry weight (DW) was recorded. The calculation formula is as follows:

(4) LRWC (%)=[(FW−DW)/(SW−DW)]×100,

(5) NWSD (%)=100−LRWC (%).

### 4.5. Soluble Sugar and Amino Acid Content in the Leaf

The contents of soluble sugar and amino acid in the fourth leaves were determined at 3 DAT Soluble sugar and amino acid was extracted by the ethanol-soluble extraction method, as described by Hu [31]. Soluble sugar and amino acid were determined by anthrone colorimetry [30] and ninhydrin solution detection [32,33], respectively.

### 4.6. Photosynthetic Pigment Content of Leaves

Photosynthetic pigments (chlorophyll a (Chl a), chlorophyll b (Chl b), total chlorophyll (T Chl), and carotenoid (Car)) were determined using a spectrophotometer (UV-1280, Shimadzu, Kyoto, Japan) [34]. In short, a fresh leaf of 0.05 g was weighed and placed in a 10 mL centrifuge tube containing 8 mL, 95% ethanol in the dark for 48 h. The tube was vibrated during the extraction process. OD values at 470 nm, 649 nm, and 665 nm wavelengths were recorded.

### 4.7. Photosynthetic Parameters of Leaves

The net photosynthetic rate (Pn), transpiration rate (Tr), stomatal conductance (Gs) and intercellular CO_2_ concentration (Ci) of the fourth true leaf were determined by portable photosynthetic apparatus (Li-6800, LI-COR, Lincoln, NE, USA). Photosynthetic parameters were set as follows: humidity, 55 ± 5%; CO_2_, 400 ± 5 μmol mol^−1^; photosynthetic intensity, 180–200 μmol m^−2^ s^−1^.

### 4.8. Data Processing and Analysis

#### 4.8.1. Comprehensive Evaluation of MFV by Principal Component Weighting Analyses

The method of principal component weighting was firstly used to carry out a comprehensive evaluation analysis of MFV.

(1) Weight of indexes by PCA:
  (6) W=Σ[PC(m)×I(jm)].
  PC(m) is the variance contribution degrees of the m-eth principal component. I(jm) indicate the eigenvalue of the j-eth index in the m-eth principal component. W is the weight (Wj) of the j-eth index after normalization.
(2) MFV calculation in fuzzy mathematics:
  (7) Vija=(Xij−Xjmin)/(Xjmax−Xjmin),
  (8) Vijb=1−(Xij−Xjmin)/(Xjmax−Xjmin).
  Vija and Vijb refer to the membership function value of index j treated by TDZ. Moreover, Vija and Vijb indicate the positive and negative correlations with the sensitivity of cotton leaf shedding, respectively. Xij represents the measured mean value of the index under different TDZ concentrations. Xjmax and Xjmin are the maximum and minimum values of j index, respectively.

The final membership function value (MV) of comprehensive evaluation:

(9) MVi=Σ(Vij×Wj).

Leaf abscission sensitivity was assessed by the value of the final membership function (MV). The higher the MV, the stronger the leaf abscission sensitivity is.

#### 4.8.2. Correlation Analysis

(1) The Pearson correlation between 13 physiological indexes and the MV was analyzed.
(2) The correlation of all morpho-physiological characters was analyzed by Pearson correlation analysis.

#### 4.8.3. Variance Analysis and Cluster Analysis

Origin Pro2018 was used for data analysis and plotting.

## 5. Conclusions

In conclusion, CRI 12 was more sensitive than CRI 49 to leaf abscission induced by TDZ. The concentration of 100 mg L^−1^ TDZ could be used as the optimal concentration for the shedding of cotton leaves. Photosynthetic parameters (Pn, Tr, and Gs) were reliable indexes to evaluate the cotton leaf shedding sensitivity. Furthermore, we proposed a mathematical evaluation model to assess the cotton leaf abscission sensitivity that could be potentially used to evaluate various stress responses of plants.

## Figures and Tables

**Table 1:** Weight determination by principal components analysis method.

PCA	PC 1	PC 2	PC 3	PC 4	PC 5	W
Proportion of Variance	0.51	0.22	0.08	0.05	0.05	-
Cumulative Proportion	0.51	0.73	0.82	0.87	0.91	-
AR 3	−0.12	0.03	−0.01	−0.02	−0.01	0.12
AR 4	−0.12	0.01	−0.01	−0.02	−0.01	0.13
AR 5	−0.13	0.01	−0.02	−0.01	−0.01	0.14
AR 8	−0.14	0.02	−0.02	0.00	0.00	0.12
AR 9	−0.14	0.03	−0.01	0.00	0.01	0.09
AR 10	−0.14	0.03	−0.01	0.00	0.00	0.10
Chl a	−0.03	−0.09	−0.01	−0.01	0.00	0.13
Chl b	−0.04	−0.10	−0.01	−0.01	0.00	0.14
Car	−0.12	−0.05	0.01	0.00	0.00	0.14
T Chl	−0.04	−0.10	−0.01	−0.01	0.00	0.13
Chl a/b	0.08	0.07	0.00	−0.01	0.00	−0.12
LRWC	0.10	0.05	0.02	−0.02	0.00	−0.13
NWSD	−0.10	−0.05	−0.02	0.02	0.00	0.13
SS	0.10	0.00	−0.02	0.01	0.01	−0.09
AA	−0.02	−0.06	0.04	0.00	−0.01	0.04
WI	−0.14	0.01	0.02	0.00	0.00	0.09
PSI	−0.14	0.02	0.02	0.01	0.01	0.08
Tr	0.14	−0.02	−0.02	0.00	0.00	−0.09
Pn	0.14	−0.02	−0.02	0.00	−0.01	−0.09
Ci	−0.02	0.03	−0.01	0.02	−0.04	0.02
Gs	0.14	−0.02	−0.02	0.00	0.00	−0.09

Note: AR3, AR4, AR5, AR8, AR9, AR10 represent leaf abscission rate of treatment after 3, 4, 5, 8, 9, and 10 days, respectively; Chl a, Chlb., T Chl, Car, Chl a/b, LRWC, NWSD, SS, AA, WI, PSI, Tr, Pn, Ci, Gs represent chlorophyll a, chlorophyll b, total chlorophyll, carotenoid, the ratio of chlorophyll a and chlorophyll b, leaf relative water content, natural water-saturated deficit, soluble sugar, amino acid, wilting incidence, purple spot incidence, transpiration rate, net photosynthetic rate, intercellular CO_2_ concentration, and stomatal conductance, respectively.

**Table 2:** Leaf abscission sensitive evaluation by membership function value (MFV) method.

Index	CRI 49							CRI 12						
	C0	C1	C2	C3	C4	C5	C6	C0	C1	C2	C3	C4	C5	C6
AR3	0.00	0.06	0.06	0.05	0.10	0.04	0.03	0.00	0.06	0.05	0.08	0.11	0.12	0.07
AR4	0.00	0.05	0.04	0.04	0.07	0.02	0.03	0.00	0.07	0.08	0.06	0.07	0.13	0.09
AR5	0.00	0.07	0.05	0.03	0.09	0.03	0.05	0.00	0.11	0.09	0.10	0.14	0.12	0.10
AR8	0.00	0.10	0.06	0.06	0.08	0.03	0.07	0.00	0.11	0.12	0.10	0.12	0.08	0.10
AR9	0.00	0.08	0.06	0.06	0.08	0.03	0.05	0.00	0.08	0.08	0.08	0.09	0.05	0.06
AR10	0.00	0.08	0.06	0.07	0.09	0.03	0.06	0.00	0.09	0.08	0.10	0.10	0.06	0.07
Chl a	0.11	0.11	0.12	0.13	0.12	0.06	0.11	0.08	0.12	0.10	0.10	0.06	0.10	0.00
Chl b	0.12	0.12	0.13	0.14	0.13	0.08	0.12	0.10	0.13	0.12	0.12	0.08	0.12	0.00
Car	0.00	0.07	0.04	0.07	0.05	0.08	0.07	0.02	0.05	0.04	0.07	0.10	0.04	0.14
T Chl	0.11	0.11	0.12	0.13	0.12	0.07	0.11	0.09	0.12	0.11	0.11	0.07	0.11	0.00
Chl a/b	−0.10	−0.09	−0.12	−0.08	−0.05	−0.08	−0.09	−0.11	−0.07	−0.11	−0.08	−0.09	−0.11	0.00
LRWC	−0.02	−0.06	−0.05	−0.02	−0.01	−0.02	−0.03	0.00	−0.09	−0.06	−0.04	−0.06	−0.02	−0.13
NWSD	0.02	0.06	0.05	0.02	0.01	0.02	0.03	0.00	0.09	0.06	0.04	0.06	0.02	0.13
SS	−0.04	−0.01	−0.05	0.00	−0.05	−0.02	−0.05	−0.09	−0.03	−0.05	0.00	−0.02	−0.01	−0.02
AA	0.02	0.02	0.03	0.01	0.02	0.01	0.02	0.03	0.03	0.03	0.02	0.04	0.02	0.00
WI	0.00	0.07	0.08	0.09	0.08	0.09	0.07	0.00	0.09	0.09	0.09	0.08	0.08	0.09
PSI	0.00	0.07	0.07	0.08	0.07	0.07	0.07	0.00	0.07	0.08	0.07	0.06	0.06	0.08
Tr	−0.01	−0.08	−0.08	−0.08	−0.08	−0.08	−0.08	0.00	−0.08	−0.08	−0.08	−0.08	−0.09	−0.08
Pn	−0.01	−0.09	−0.08	−0.08	−0.08	−0.09	−0.09	0.00	−0.08	−0.08	−0.08	−0.08	−0.09	−0.09
Ci	0.00	0.00	0.01	0.00	0.01	0.00	0.01	0.00	0.01	0.00	0.02	0.00	0.01	0.01
Gs	−0.01	−0.08	−0.08	−0.08	−0.08	−0.08	−0.09	0.00	−0.09	−0.08	−0.08	−0.08	−0.09	−0.08
MV	0.19	0.67	0.53	0.63	0.77	0.29	0.46	0.12	0.78	0.67	0.81	0.77	0.70	0.52

Note: MV is the value of the final membership function that can access the leaf abscission sensitivity. AR3, AR4, AR5, AR8, AR9, AR10 represent leaf abscission rate of treatment after 3, 4, 5, 8, 9, and 10 days; Chl a, Chlb., T Chl, Car, Chl a/b, LRWC, NWSD, SS, AA, WI, PSI, Tr, Pn, Ci, Gs represent chlorophyll a, chlorophyll b, total chlorophyll, carotenoid, the ratio of chlorophyll a and chlorophyll b, leaf relative water content, natural water-saturated deficit, soluble sugar, amino acid, wilting incidence, purple spot incidence, transpiration rate, net photosynthetic rate, intercellular CO_2_ concentration, and stomatal conductance, respectively.

**Table 3:** Pearson’s correlation analysis between physiological indexes and MV.

Index	Correlation	*p*-Value	Location of Correlation
Chl a	−0.271	0.349	10
Chl b	−0.183	0.530	13
Car	0.352	0.217	5
T Chl	−0.240	0.408	11
Chl a/b	−0.200	0.492	12
LRWC	−0.343	0.230	6
NWSD	0.343	0.230	7
SS	−0.553 *	0.040	4
AA	−0.266	0.358	9
Tr	−0.769 **	0.001	2
Pn	−0.760 **	0.002	3
Ci	0.291	0.313	8
Gs	−0.770 **	0.001	1

Note: * and ** represent that correlation is significant at the 0.05 and 0.01 level, respectively. Chl a, Chlb.,T Chl, Car, Chl a/b, LRWC, NWSD, SS, AA, Tr, Pn, Ci, Gs represent chlorophyll a, chlorophyll b, total chlorophyll, carotenoid, the ratio of chlorophyll a and chlorophyll b, leaf relative water content, natural water-saturated deficit, soluble sugar, amino acid, transpiration rate, net photosynthetic rate, intercellular CO_2_ concentration, and stomatal conductance, respectively.

**Table 4:** Leaf abscission rate between CRI 49 and CRI 12 in the field.

Provinces	Cultivars	CK	TDZ	Relative Leaf Abscission Rate
Xinjiang	CRI 12	49.0 b	74.1 a	1.5
	CRI 49	63.3 b	71.3 a	1.1
Henan	CRI 12	21.6 b	83.0 a	3.8
	CRI 49	35.5 b	81.4 a	2.3

Note: The different letters within the same row are significantly different at *p* = 0.05.

## Data Availability

The data presented in this study are available on request from the corresponding author.

## Supplementary Materials

The following are available online at https://www.mdpi.com/2223-7747/10/1/49/s1, Figure S1: Effects of different concentration of Thidiazuron (TDZ) on leaf abscission rate between CRI 49 and CRI 12 at the seedling stage. C0, C1, C2, C3, C4, C5 and C6 represent 0, 100, 200, 300, 400, 500, 1000 mg L^−1^ TDZ treatment, respectively.
