# Supplementary material for: Evaluation of Cotton (Gossypium hirsutum L.) Leaf Abscission Sensitivity Triggered by Thidiazuron through Membership Function Value
Source: Plants (Basel). 2020 Dec 28;10(1):49. doi: 10.3390/plants10010049 (PMC7823538; doi:10.3390/plants10010049)
Supplement: Supplementary file 1 [file plants-10-00049-s001.pdf]

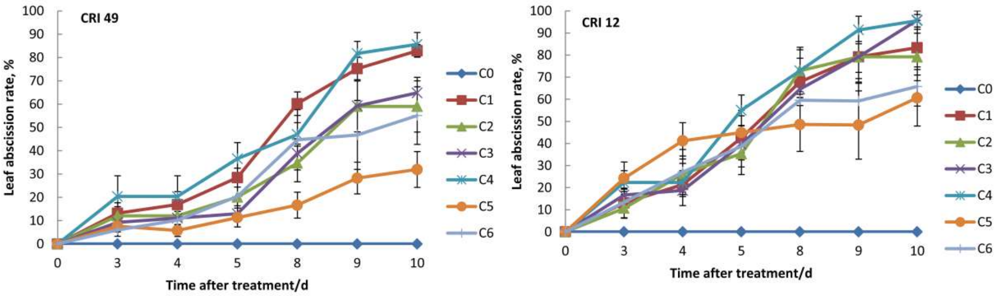

Figure S1. Effects of different concentration of Thidiazuron (TDZ) on leaf abscission rate between CRI 49 and CRI 12 at the seedling stage. C0, C1, C2, C3, C4, C5 and C6 represent 0, 100, 200, 300, 400, 500, 1000 mg L^{-1} TDZ treatment, respectively.
